# Study of P wave dispersion in patients with paroxysmal atrial fibrillation and its role in prediction of atrial fibrillation recurrence

**DOI:** 10.1186/s43044-024-00503-4

**Published:** 2024-06-27

**Authors:** Mohammed Magdy Mohammed Gomaa, Eman Elsayed Ali Elsafty, Hend Magdy Mohamed Gomaa, Mona Malek Abdulrahim, Ahmed Hassan Hosny Eladawy

**Affiliations:** Faculty of Medicine, Mansoura University, Mansoura, Egypt

**Keywords:** P wave dispersion, Paroxysmal atrial fibrillation, Recurrence

## Abstract

**Background:**

It has been known that increased P wave duration and P wave dispersion reflect prolongation of intra-atrial and interatrial conduction time and the inhomogeneous propagation of sinus impulses, which are well-known electrophysiologic characteristics in patients with atrial arrhythmias and especially paroxysmal atrial fibrillation. The objective of this study was assessment of P wave dispersion value in cases with paroxysmal atrial fibrillation and its role in predicting recurrence.

**Results:**

Forty-eight patients with documented paroxysmal AF were subjected to clinical evaluation, electrocardiogram and routine Doppler echocardiogram. We found that a statistically significant association was detected between P wave dispersion and older age, diabetic and hypertensive cases with positive correlation also detected with left atrial dimension (LAD), left ventricle size and diastolic dysfunction grade. Mean corrected P wave dispersion and corrected QT interval were higher among cases using sotalol, ca channel blockers, among cases using nitrates and among cases with Morris index > 0.04. Higher mean value of corrected QT was associated with biphasic P v1 shape. Old age, female sex, P wave dispersion and QT wave dispersion are statistically significant predictors of PAF recurrence.

**Conclusion:**

P wave dispersion in patients with paroxysmal atrial fibrillation was strongly correlated to older age, diabetic and hypertensive patients and also with left atrial dimension (LAD), left ventricle size and diastolic dysfunction grade. Also, mean corrected P wave dispersion can predict atrial fibrillation recurrence in patients with Morris index > 0.04, old age, female sex, and QT wave dispersion.

## Background

Atrial fibrillation (AF) is the most commonly treated arrhythmia. Its prevalence in the population increases with age, and it is estimated to affect over 4 percent of the population above the age of 60. PAF is defined as AF that terminates spontaneously or with intervention within seven days of onset. PAF has been reported as comprising 25–62% of AF cases. The prevalence of PAF may be underestimated, as many episodes (including some lasting more than 48 h) are asymptomatic. Also, the duration of recurrent AF episodes varies over time in each individual, and progression to persistent or permanent AF is common [1].

Atrial fibrillation is associated with electrical and structural myocardial remodeling and autonomic dysregulation of the heart which should be reflected in increased electrocardiogram (ECG) signal variability. However, changes to ECG characteristics, such as P wave morphology or heart rate variation, are generally poorly associated with AF incidence and consequent stroke, especially for prediction of PAF. However, P wave axis variation is a reasonable predictor and supports the concept that small variations of the sinus rhythm ECG waveform might be useful to predict PAF [2].

P wave dispersion (PWD, Pd or Pdis) is a noninvasive electrocardiographic (ECG) marker for atrial remodeling and predictor for atrial fibrillation (AF). PWD is defined as the difference between the widest and the narrowest P wave duration recorded from the 12 ECG leads. Increased P wave duration and PWD reflect prolongation of intra-atrial and interatrial conduction time with lack of a well-coordinated conduction system within the atrial muscles [3]. Many studies suggested the presence of significant correlation between P wave dispersion and atrial fibrillation [4].

P wave dispersion is a valuable tool for predicting atrial fibrillation, and it could support means to control several risk factors of atrial fibrillation and prevent its devastating clinical outcome [5].

## Methods

This is a cross-sectional study with comparative component that was carried out on 48 patients with documented paroxysmal AF from Cardiology Department, Specialized Internal Medical Hospital, Mansoura University, from June 2020 to June 2022.

All patients were monitored after restoring the sinus rhythm by attending physically in outpatient clinic for arrhythmia and OAC in Mansoura specialized medical hospital every month for 12 months, and trans-telephonic if any attacks of palpitations or other symptoms happened to our patients in between the clinic visits.

### Sample size calculation

Sample size calculation was based on difference in mean P wave dispersion between cases with and without PAF retrieved from previous research [6].

Using G power program version 3.1.9.4 to calculate sample size based on effect size of 0.96, using 2-tailed test, *α* error = 0.05 and power = 90.0%, the total calculated sample size was estimated to be 48 cases with the following inclusion criteria patients with documented paroxysmal AF (one or more attacks) after restoration of normal sinus rhythm. Patients with congenital heart disease, permanent AF, pacemaker, congenital defects and undetected P wave were excluded from this study.

Patients after termination of paroxysmal AF episode rhythm were interrogated about the study, and those who agreed to participate were subjected to:

- Clinical evaluation by assessment of sociodemographic characteristics, medical history, current complaints, detailed history of PAF dysrhythmia including precipitating factors, duration of episode, method of termination of last attack and current medication history. Also, general and local cardiac examination was done
- Revision and documentation of laboratory investigations; CBC, random blood sugar, serum creatinine, CRP and thyroid hormones.
- Electrocardiogram (ECG): Full 12 leads were done for every patient at least one week after the restoration of normal sinus rhythm (all data were reviewed by 3 doctors at different times) with assessment of the following (ECG recording was done by adjusting the instrument setting to record 5 cycles in each lead): P wave amplitude, P wave duration, PR interval, QRS duration, QT interval (corrected), morphology (smooth, biphasic, monophasic), RR duration and calculation of heart rate (bpm), QRS duration, QT duration (sec) and corrected QT interval (QTc) using Bazett formula [7], QRS axis (degree) using the quadrant and super-SAM the Axis Man methods [8], ST segment changes (elevation or depression), duration of PR segment in Lead II, P wave in Lead II (shape duration and amplitude), P wave in V1 for shape (monophasic/biphasic), duration (sec) and amplitude (mm), duration and amplitude of negative component of Pv1 and calculation of Morris index (P wave terminal force) = amplitude × duration of the terminal negative portion of the P wave in lead V1 (when area exceeded 0.04 mm sec indicates left atrial enlargement) [9].
- Routine Doppler echocardiogram: M-Mode and two-dimensional (2D) and Doppler echocardiographic examination were performed for all patients using the echocardiographic apparatus GE Vivid 7 with 2.5 and 3.5 MHZ transducers. All examinations were done one week after restoring normal sinus rhythm. All examinations were performed with patient in left lateral decubitus position in accordance with the recommendation of American Society of Echocardiography [10].

### Ethical consideration

The study protocol was approved by IRB committee in faculty of medicine, Mansoura University, after obtaining informed verbal consent from every patient sharing in the study after confirming that data collected from patients were not used in other purposes rather than the present research.

### Statistical analysis

Data were collected and tabulated. For data analysis, different grouping was assumed according to patient demographic and clinical parameters, duration of PAF disease as well as number of PAF episodes. Analyzed data were done using SPSS (statistical package for social sciences) version 22. Qualitative data were presented as number and percent, and quantitative data were tested for normality by Kolmogorov–Smirnov test and then described as mean and standard deviation for normally distributed data and median and range for non-normally distributed. The appropriate statistical tests were applied according to data type with the following suggested tests: Student’s t test and one-way ANOVA test were used to compare more than two independent groups with post hoc Tukey test to detect pair-wise comparison for continuous variables. The Spearman’s rank-order correlation was used to determine the strength and direction of a linear relationship between two non-normally distributed continuous variables and/or ordinal variables. Binary logistic regression was used to detect significant predictors of recurrence with calculation of adjusted odds ratio. Receiver operating characteristic curve was used to detect best cutoff point for P wave dispersion in prediction of recurrence.

## Results

**Fig. 1:**
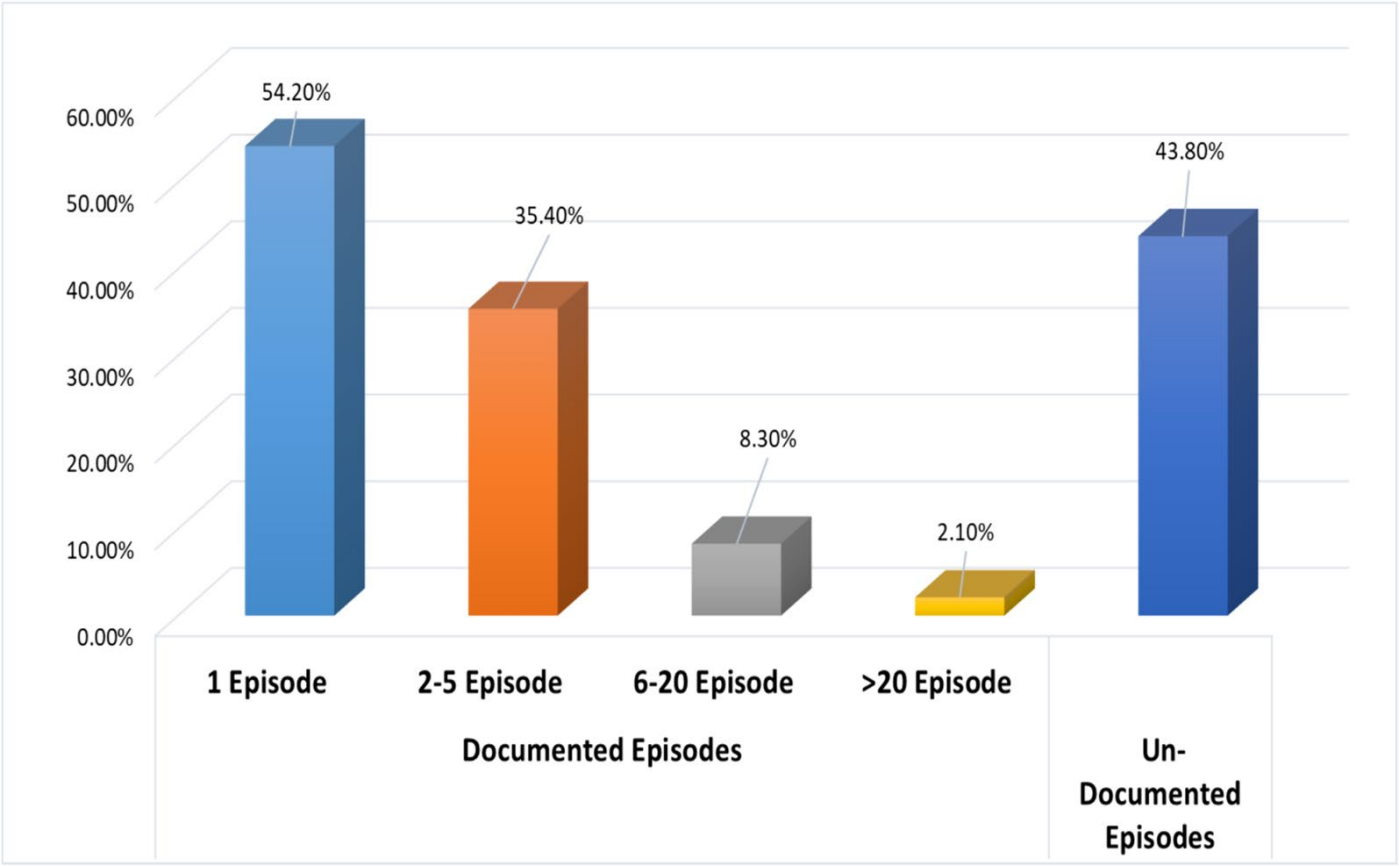
Number of episodes of PAF among studied cases

**Fig. 2:**
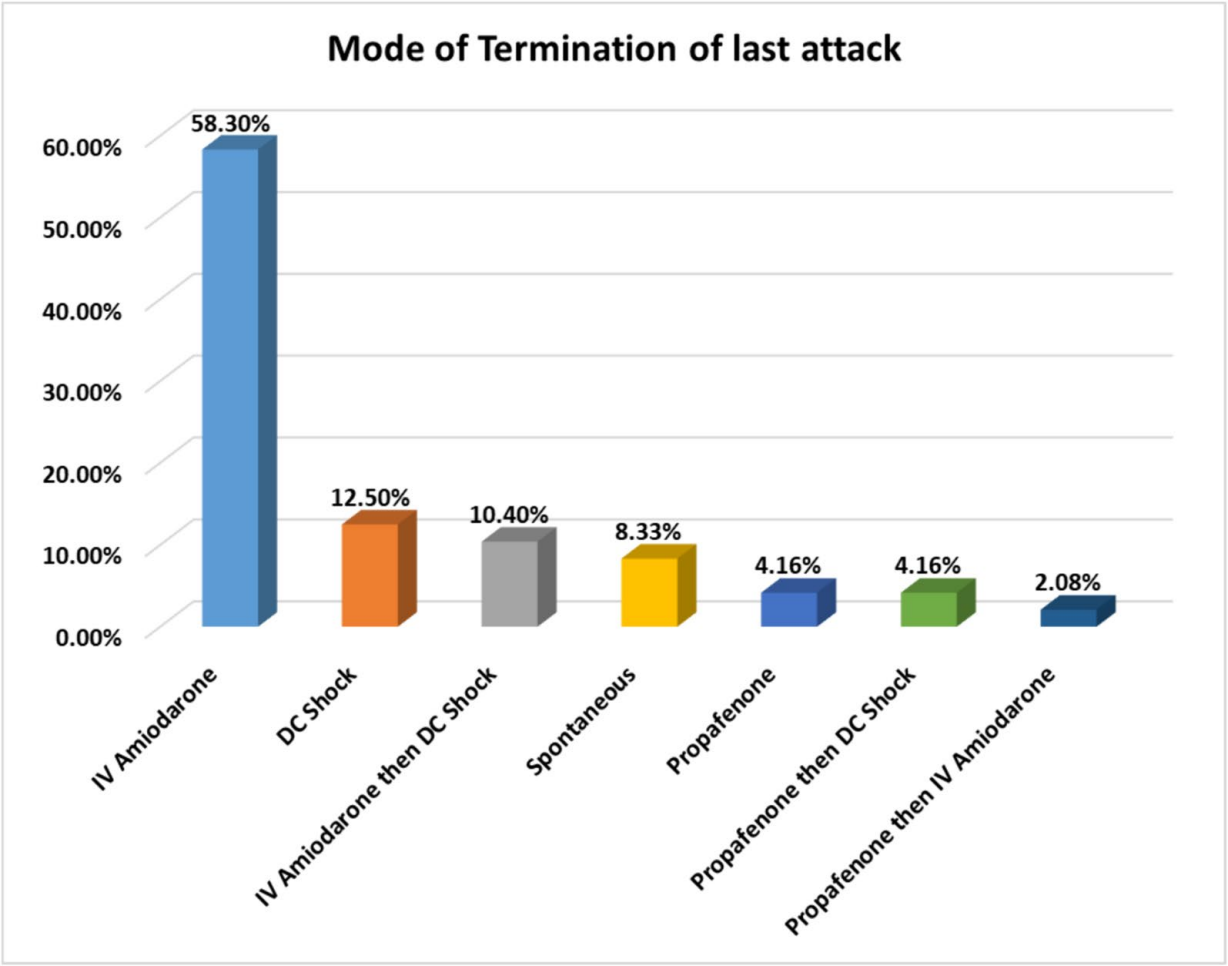
Mode of termination of last PAF attack

The present study was carried out on 48 PAF cases with their mean age (SD) which was 52.21 (12.43) years ranging from 22 to 81 years, and mean age (SD) of AF onset was 50.69 (12.62) years ranging from 22 to 81 years, 58.3% females, 64.6% urban residence, 41.7% housewives, 25% manual worker, 27.1% office worker and 6.2% retired. Of the studied cases, 39.6% were secondary education, 27.1% illiterate, 22.9% postgraduate and 10.4% primary education. As regards marital status, 93.8% are married, 2.1% are single and 4.2% are divorced. Health care provision was done through insurance for 29.2% of the cases and 70.8% non-insured. Of the studied cases, 6.2% were on sotalol, 2.1% propafenone, 18.8% beta blocker, other 12.5% CCB, 4.2% apixaban, 6.2% warfarin, 4.2% clopidogrel, 2.1% acetyls, 16.7% diuretics, 31.2% ACEI/ACEB, 8.3% nitrates, 2.1% trimetazidine, 6.2% statins, 18.8% oral hypoglycemic, 4.2% insulin and 2.1% carbimazole. Mean PAF duration is 3.64 ± 5.42 years ranging from 1 month to 25 years. All documented attacks were distributed as follows: 54.2% only one episode and 45.8% more than one episode. Among studied cases, 43.8% have associated undocumented AF episodes as shown in Fig. 1. Mode of termination of last episode was as follows: 8.3% spontaneous, 4.2% propafenone, 2.1% propafenone and then IV amiodarone, 4.2% propafenone and then DC Shock, 10.4% IV amiodarone and then DC Shock, IV amiodarone, 58.3% IV amiodarone and 12.5% DC shock as shown in Fig. 2.

A statistically significant relation was detected between P wave dispersion and older age of the studied cases and also while classifying sex by age group demonstrates also statistically significant higher mean dispersion among older age group and among cases with older age at AF onset. Higher mean P wave dispersion and corrected P wave dispersion were detected among diabetes cases and hypertensive cases as shown in Table 1

**Table 1:** Relation between p wave dispersion, corrected p wave dispersion and demographic characteristics among studied PAF patients

	P wave dispersion		Corrected P wave dispersion	
	(Mean ± SD)	significance	(Mean ± SD)	significance
*Age at PAF presentation (years) total cases*				
20–40 (*n* = 7)	0.038 ± 0.004^AB^	*F* = 11.09	0.040 ± 0.005^AB^	*F* = 10.41
> 40–60 (*n* = 29)	0.078 ± 0.023^A^	*p* < 0.001*	0.085 ± 0.027^A^	*p* < 0.001*
≥ 60 (*n* = 12)	0.083 ± 0.026^B^		0.088 ± 0.024^B^	
*Age at PAF presentation (years) among males*				
20–40 (*n* = 2)	0.036 ± 0.006^AB^	*F* = 4.45	0.0037 ± 0.009^A^	*F* = 3.18
> 40–60 (*n* = 13)	0.08 ± 0.02^A^	*p* = 0.028*	0.0915 ± 0.028^A^	*p* = 0.07
≥ 60 (*n* = 5)	0.078 ± 0.014^B^		0.087 ± 0.028	
*Age at PAF presentation (years) among females*				
20–40 (*n* = 5)	0.038 ± 0.004^AB^	*F* = 6.24	0.042 ± 0.004^AB^	*F* = 7.26
> 40–60 (*n* = 16)	0.077 ± 0.025^A^	*p* = 0.006*	0.081 ± 0.026 ^A^	*p* = 0.003*
≥ 60 (*n* = 7)	0.086 ± 0.028^B^		0.09 ± 0.023^B^	
*Age of AF onset among total cases(years)*				
20–40 (*n* = 8)	0.043 ± 0.015 ^AB^	*F* = 8.83	0.044 ± 0.01 ^AB^	*F* = 10.1
> 40–60 (*n* = 30)	0.078 ± 0.02 ^A^	*p* = 0.001*	0.086 ± 0.026 ^A^	*p* < 0.001*
≥ 60 (*n* = 10)	0.083 ± 0.02^B^		0.089 ± 0.029^B^	
*Male age at AF onset*				
20–40 (*n* = 3)	0.036 ± 0.005^AB^	*F* = 4.45	0.037 ± 0.009	*F* = 3.18
> 40–60 (*n* = 14)	0.080 ± 0.02^A^	*p* = 0.028*	0.092 ± 0.028	*p* = 0.07
≥ 60 (*n* = 3)	0.078 ± 0.014^B^		0.087 ± 0.029	
*Female age at AF onset*				
20–40 (*n* = 5)	0.038 ± 0.003^AB^	*F* = 6.24	0.042 ± 0.004 ^AB^	*F* = 7.26
> 40–60 (*n* = 16)	0.077 ± 0.025^A^	*p* = 0.006*	0.081 ± 0.026 ^A^	*p* = 0.003*
≥ 60 (*n* = 7)	0.086 ± 0.027^B^		0.09 ± 0.023 ^B^	
*Health care provision*				
Insurance (*n* = 14)	0.073 ± 0.023	*t* = 0.087	0.083 ± 0.029	*t* = 0.464
No insurance (*n* = 34)	0.074 ± 0.028	*p* = 0.931	0.079 ± 0.029	*p* = 0.645
*History of diabetes*				
Negative (*n* = 37)	0.068 ± 0.02	*t* = 2.58	0.075 ± 0.024	*t* = 2.22
Positive (*n* = 11)	0.090 ± 0.036	*p* = 0.013*	0096 ± 0.038	*p* = 0.032*
*History of hypertension*				
Negative (*n* = 24)	0.058 ± 0.020	*t* = 4.58	0.064 ± 0.024	*t* = 4.42
Positive (*n* = 24)	0.087 ± 0.023	*p* < 0.001*	0.095 ± 0.024	*p* < 0.001*
*Smoking history*				
Non-smoker (*n* = 43)	0.074 ± 0.027	*t* = 0.945	0.08 ± 0.03	*t* = 0.859
Smoker (*n* = 5)	0.08 ± 0.0	*p* = 0.396	0.087 ± 0.007	*p* = 0.430

*t* Student’s t test, F: One-way ANOVA test, p: probability, similar superscripted letter denotes significant difference between groups within same column

*Statistically signficiant difference based on *p* value < 0.05

A statistically significant positive correlation was also detected between P wave dispersion and the following: LAD (*r* = 0.414), left ventricle size (*r* = 0.379), diastolic dysfunction grade (*r* = 0.354). Also, a statistically significant positive correlation was detected between corrected P wave dispersion and the following: LAD (*r* = 0.386), LV size (*r* = 0.407) and diastolic dysfunction grade (*r* = 0.413). However, a statistically significant negative correlation was detected between corrected P wave dispersion and the presence of aortic cusps sclerosis (*r* = 0.330). A statistically significant positive correlation was detected between PR duration and LAD (*r* = 0.334) and also between P wave axis and the following: LVH (*r* = 0.296), diastolic dysfunction grade (*r* = 0.291), negative correlation with presence of aortic cusp sclerosis (*r* = − 0.336) as shown in Table 2.

**Table 2:** Correlation between P wave dispersion, corrected P wave dispersion and Doppler EHO findings among studied PAF patients

	p wave dispersion		Corrected p wave dispersion		PR duration		P wave axis		Morris Index	
	*r*	*p* value	*r*	*p* value	*r*	*p*	*r*	*p*	*r*	*p*
LVEDD (cm)	0.254	0.081	0.223	0.128	− 0.108	0.463	0.111	0.454	− 0.117	0.43
LVESD (cm)	0.116	0.431	0.081	0.583	− 0.272	0.062	0.113	0.442	− 0.1	0.499
EF (ratio)	− 0.01	0.944	0.02	0.894	0.217	0.138	0.099	0.053	0.113	0.445
FS (%)	0.039	0.793	0.064	0.666	0.191	0.192	0.07	0.656	0.128	0.386
IVST (cm)	0.161	0.274	0.131	0.376	0.104	0.481	0.201	0.17	0.021	0.89
PWT (cm)	0.105	0.47	0.135	0.36	0.183	0.213	0.175	0.234	0.031	0.834
ARD (cm)	0.253	0.083	0.237	0.105	− 0.036	0.807	0.211	0.15	− 0.134	0.365
LAD (cm)	0.414	0.003*	0.386	0.007*	0.334	0.02*	0.125	0.399	0.104	0.483
LA size	0.173	0.24	0.159	0.28	− 0.009	0.952	0.009	0.95	− 0.068	0.644
LV size	0.379	0.008*	0.407	0.004*	0.066	0.655	0.296	0.04*	0.01	0.941
2D MV	− 0.103	0.486	− 0.127	0.388	− 0.268	0.07	− 0.05	0.734	− 0.143	0.332
Diastolic dysfunction grade	0.354	0.014*	0.413	0.004*	0.156	0.29	0.291	0.045*	− 0.18	0.22
Mitral Regurgitation	0.182	0.216	0.193	0.189	− 0.098	0.509	0.157	0.286	− 0.028	0.849
Aortic Cusps sclerosis	0.055	0.713	0.075	0.611	− 0.188	0.2	− 0.336	0.02*	0.169	0.251
Aortic Regurge	− 0.284	0.051	− 0.33	0.02*	0.071	0.634	0.123	0.403	0.072	0.625
Tricuspid Regurge	0.113	0.445	0.031	0.835	0.145	0.324	0.209	0.154	0.006	0.966

*Statistically signficiant difference based on *p* value < 0.05

A statistically signficant relation was detected between value of corrected p wave dispersion, corrected QT and using anticoagulant and Ca channel blockers.Higher value of mean corrected QT was detected among cases using anti-coagulation before restoration of NSR with statistically significant association. Corrected p wave dispersion was significiantly associated with use of nitrates (Table 3).

**Table 3:** Association between p wave dispersion, corrected P wave dispersion, corrected QT interval and medications used among studied patients

	P wave dispersion		Corrected P wave dispersion		Corrected QT Interval	
	Mean ± SD	Significance	Mean ± SD	Significance	Mean ± SD	Significance
*Sotalol*						
Not used	0.070 ± 0.02	*t* = 3.57	0.076 ± 0.027	*t* = 3.11	0.416 ± 0.03	*t* = 2.09
Used	0.120 ± 0.0	*p* = 0.001*	0.126 ± 0.005	*p* = 0.003*	0.458 ± 0.06	*p* = 0.04*
*BB other than Sotalol*						
Not used	0.072 ± 0.02	*t* = 0.557	0.078 ± 0.03	*t* = 0.684	0.420 ± 0.03	*t* = 0.753
Used	0.077 ± 0.016	*p* = 0.580	0.086 ± 0.022	*p* = 0.498	0.411 ± 0.025	*p* = 0.355
*CCB*						
Not used	0.07 ± 0.02	*t* = 2.46	0.076 ± 0.025	*t* = 2.63	0.414 ± 0.02	*t* = 2.44
Used	0.096 ± 0.04	*p* = 0.018*	0.108 ± 0.04	*p* = 0.01*	0.449 ± 0.05	*p* = 0.02*
*Anti-coagulation before restoration of NSR*						
Not used	0.072 ± 0.024	*t* = 1.11	0.078 ± 0.028	*t* = 0.779	0.415 ± 0.03	*t* = 2.49
Used	0.085 ± 0.04	*p* = 0.273	0.089 ± 0.038	*p* = 0.440	0.453 ± 0.038	*p* = 0.02*
*Diuretics used*						
Not used	0.071 ± 0.02	*t* = 1.46	0.077 ± 0.02	*t* = 1.41	0.416 ± 0.03	*t* = 1.32
Used	0.086 ± 0.04	*p* = 0.152	0.093 ± 0.048	*p* = 0.165	0.433 ± 0.045	*p* = 0.195
*ACEI/ACEB*						
not used	0.066 ± 0.01	*t* = 2.86	0.077 ± 0.02	*t* = 1.41	0.416 ± 0.03	*t* = 1.32
used	0.088 ± 0.04	*p* = 0.006*	0.093 ± 0.048	*p* = 0.165	0.433 ± 0.045	*p* = 0.195
*Nitrates*						
Not used	0.07 ± 0.02	*t* = 3.21	0.077 ± 0.027	*t* = 2.76	0.416 ± 0.032	*t* = 1.71
Used	0.11 ± 0.02	*p* = 0.002*	0.116 ± 0.028	*p* = 0.008*	0.446 ± 0.053	*p* = 0.09
*Statins*						
Not used	0.072 ± 0.02	*t* = 1.38	0.079 ± 0.029	*t* = 0.985	0.418 ± 0.04	*t* = 0.283
Used	0.093 ± 0.023	*p* = 0.174	0.096 ± 0.012	*p* = 0.330	0.424 ± 0.03	*p* = 0.779
*Antidiabetic SV*						
Negative	0.075 ± 0.028	*t* = 0.722	0.08 ± 0.03	*t* = 0.194	0.419 ± 0.037	*t* = 0.399
Positive	0.068 ± 0.02	*p* = 0.474	0.078 ± 0.02	*p* = 0.847	0.415 ± 0.027	*p* = 0.692

*t* Student’s *t* test

^*^statistically significant

As regards in relation to ECG finding, higher mean P wave dispersion and corrected P wave dispersion were detected among cases with Morris index more than 0.04, and also, a statistically significant positive correlation is detected between P wave dispersion and QT interval (*r* = 0.325) and corrected QT wave (*r* = 0.333) and also between corrected P wave dispersion with maximum heart rate (*r* = 0.312), mean heart rate (*r* = 0.301), QT interval (*r* = 0.312) and corrected QT interval (*r* = 0.354) as shown in Table 4.

**Table 4:** Associations between P wave dispersion, corrected P wave and ECG finding among studied patients

	P wave dispersion		Corrected P wave dispersion	
	Mean ± SD	Significance	Mean ± SD	Significance
PR interval (s)**				
Normal	0.0719 ± 0.02	*t* = 1.34	0.078 ± 0.02	*t* = 0.761
Prolonged	0.09 ± 0.02	*p* = 0.186	0.091 ± 0.02	*p* = 0.450
*Corrected QT interval*				
Normal	0.08 ± 0.0	*t* = 0.254	0.092 ± 0.0	*t* = 0.419
Abnormal	0.073 ± 0.03	*p* = 0.801	0.079 ± 0.029	*p* = 0.677
*QRS Axis (degree)*				
Normal Axis	0.074 ± 0.025	*t* = 0.347	0.0811 ± 0.027	*t* = 0.363
Left Axis	0.071 ± 0.03	*p* = 0.730	0.077 ± 0.04	*p* = 0.719
*Ventricular hypertrophy*				
No RVH	0.074 ± 0.025	*t* = 0.961	0.081 ± 0.02	*t* = 0.741
RVH	0.056 ± 0.03	*p* = 0. 342	0.065 ± 0.03	*p* = 0.462
*PII shape*				
Normal	0.068 ± 0.02	*F* = 1.39	0.073 ± 0.022	*F* = 2.37
Mitral	0.081 ± 0.03	*p* = 0.260	0.091 ± 0.03	*p* = 0.105
P pulmonal	0.08 ± 0.028		0.09 ± 0.03	
*P v1 shape*				
Monophasic	0.066 ± 0.02	*t* = 0.663	0.071 ± 0.022	*t* = 0.686
Biphasic	0.074 ± 0.02	*p* = 0.510	0.081 ± 0.03	*p* = 0.496
Morris index****				
≤ 0.04	0.064 ± 0.02	*t* = 2.11	0.068 ± 0.022	*t* = 2.34
> 0.04	0.0796 ± 0.03	*p* = 0.04*	0.088 ± 0.03	*p* = 0.024*

	*r*	*p*	*r*	*p*
Minimum heart rate	− 0.073	0.622	0.256	0.079
Maximum heart rate	0.024	0.87	0.312	0.03*
Mean heart rate	− 0.032	0.828	0.301	0.038*
QRS duration mean	0.056	0.705	− 0.05	0.736
QT interval	0.325	0.024*	0.312	0.03*
Corrected QT interval	0.333	0.02*	0.354	0.05*
QRS axis (degree)	0.024	0.874	0.026	0.862
P wave axis	− 0.069	0.641	− 0.131	0.375
PII amplitude (mv)	0.167	0.258	0.231	0.114
Total PV1 duration	0.091	0.543	0.144	0.334
P terminal Force	0.196	0.182	0.258	0.08

*Statistically signficiant difference based on *p* value < 0.05

A statistically significant association between P v1 shape and corrected QT dispersion with higher mean value of corrected QT among cases with biphasic p v1 shape is shown in Table 5.

**Table 5:** Association between corrected QT wave and PII shape and P v1 shape and Morris index

	Corrected QT wave	
	Mean ± SD	Significance
*PII shape*		
Normal	0.415 ± 0.034	*F* = 0.855
Mitral	0.429 ± 0.038	*p* = 0.432
P pulmonal	0.410 ± 0.038	
*P v1 shape*		
Monophasic	0.388 ± 0.03	*t* = 2.17
Biphasic	0.422 ± 0.03	*p* = 0.035*
*Morris index*		
≤ 0.04	0.129 ± 0.084	*t* = 0.459
> 0.04	0.140 ± 0.085	*p* = 0.649

*Statistically signficiant difference based on *p* value < 0.05

PAF recurrence was detected among 64.6% of the studied cases, with the following factors among predictors of PAF recurrence: old age (AOR: 27.5), female sex (AOR: 5.14), P wave dispersion (AOR: 11.1) and QT wave dispersion (AOR: 9.58) with the overall % predicted from regression model that is 87.5% by the previous four models as shown in Table 6.

**Table 6:** Predictors of PAF recurrence among studied cases

Predictors	Total cases *N* = 48	Recurrent PAF attacks		Univariate analysis		Multivariate analysis	
		No recurrence *N* = 17(35.4%)	Recurrence *N* = 31(64.6%)	*p* value	COR (95%CI)	P value	AOR (95% CI)
Age of onset (years)							
20–40 (*r*)	8	6 (75.0%)	2 (25.0%)	0.03*	1	1	12.62 (0.543–17.8)
> 40–60	30	10 (33.3%)	20 (66.7%)		6 (1.02–35.3)	0.114	
≥ 60	10	1 (10.0%)	9 (90.0%)	0.004*	27 (1.98–36.8)	0.003*	27.5 (17.5–30.6)
Sex							
Male(*r*)	20	11 (55.0%)	9 (45.0%)	0.016*	4.48 (1.27–15.82)	0.04*	5.14 (2.1–10.89)
Female	28	6 (21.4%)	22 (78.6%)				
History of diabetes	11	2 (18.2%)	9 (81.8%)	0.173	3.07 (0.579–16.25)		
History of Hypertension	24	3 (12.5%)	21 (87.5%)	0.001*	9.8 (2.28–42.06)	0.09	5.8 (0.489–5.26)
Smoking history							
Non-smoker (R)	43	14 (32.6%)	29 (67.4%)	0.224	0.322 (0.048–2.15)		
Smoker	5	3 (60.0%)	2 (40.0%)				
Morris index							
< 0.04	19	6 (31.6%)	13 (68.4%)	0.653	0.755 (0.222–2.56)		
> 0.04	29	11 (37.9%)	18 (62.1%)				
PR interval (*s*)**							
Normal (*r*)	44	15 (34.1%)	29 (65.9%)	0.52	0.517 (0.07–4.05)		
Prolonged	4	2 (50%)	2 (50.0%)				
QRS axis (degree)							
Normal axis (r)	38	19 (50.0%)	19 (50%)	0.09	0.25 (0.046–1.33)		
Left axis	10	8 (80.0%)	2 (20%)				
P v1 shape							
Monophasic(r)	5	1 (20%)	4 (80%)	0.922	1.12 (0.106–11.95)		
Biphasic	43	6 (18.2%)	27 (81.8%)				
LVEDD (cm)	48	4.48 ± 0.71	4.62 ± 0.71	0.572	5.50 (0.015–7.8)		
LVESD (cm)	48	2.93 ± 0.60	2.96 ± 0.51	0.240	0.004 (0.001–40.02)		
EF (ratio)	48	65.58 ± 4.36	65.09 ± 3.63	0.938	0.972 (0.480–1.97)		
FS (%)	48	36.06 ± 3.72	35.81 ± 2.65	0.417	0.728 (0.338–1.57)		
IVST (cm)	48	0.979 ± 0.175	1.08 ± 0.23	0.707	0.215 (0.002–65.8)		
PWT (cm)	48	0.912 ± 0.14	1.01 ± 0.18	0.387	8.6 (0.005–15.9)		
ARD (cm)	48	2.92 ± 0.41	3.12 ± 0.46	0.095	7.85 (0.699–88.3)		
PAF disease duration	48	13.88 ± 24.86	21.97 ± 56.74	0.585	1.0 (0.989–1.02)		
P wave dispersion	48	0.059 ± 0.02	0.081 ± 0.025	0.01*	12.7 (9.8–15.9)	0.02*	11.1 (8.7–25.9)
P wave mean duration	48	0.083 ± 0.01	0.105 ± 0.157	0.608	8.47 (0.002–29.8)		
P wave axis	48	0.167 ± 0.027	0.158 ± 0.036	0.341	1.02 (0.982–1.05)		
QT wave dispersion	48	0.104 ± 0.08	0.137 ± 0.07	0.02*	10.25 (1.25–16.8)	0.04*	9.58 (2.4–15.8)

Overall % predicted = 87.5%

Model *χ*^2^ = 28.83

*p* < 0.001*

**Fig. 3:**
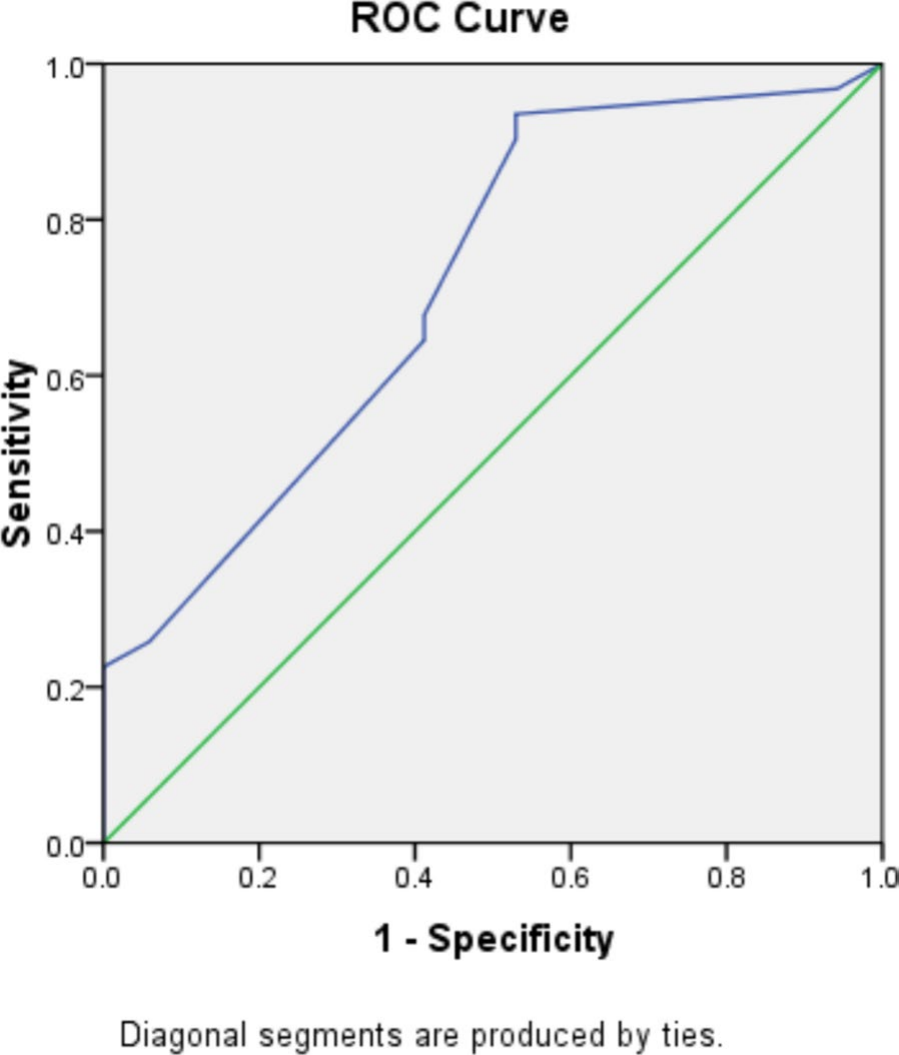
ROC curve of P wave dispersion in predicting PAF attacks among studied cases

ROC (receiver operating characteristics) curve was used to assess validity of P wave dispersion in predicting PAF recurrence and illustrates that area under curve was good (0.716; 95% CI 0.562–0.870, *p* = 0.014) with the best detected cutoff point from the curve that was 0.044 s yielding sensitivity of 93.5% and specificity 47.1% as shown in Fig. 3.

## Discussion

The current study aimed to study of P wave dispersion in patients with paroxysmal atrial fibrillation and its role in prediction of atrial fibrillation recurrence.

In the present study, there was a significant association between P wave dispersion and older age of the studied cases that was in agreement with a study on 90 cases with idiopathic PAF and reported also positive correlation between age and P wave dispersion (*r* = 0.27, *p* < 0.001) [11].

Also, significantly higher mean P wave dispersion was reported among hypertensive cases common in agreement with Ertem et al. [12] who reported that hypertension is associated with left ventricular hypertrophy, and diastolic dysfunction and increased left atrium (LA) diameter/volume are associated with PWDIS [13].

Diabetes was also associated with higher mean P wave dispersion in our study that was in agreement with the study carried out on 88 T2DM patients and reported higher P wave dispersion among cases with T2DM than non-diabetic (40.6 ± 7.6 ms vs. 33.6 ± 5.9 ms) [12].

In the present study, P wave dispersion showed statistically significant positive correlation with LAD and left ventricle size that was also in agreement with Saravi et al. [14] study [15]. Additionally, Ozyigit et al. [16] studied the relationship between PWD and left atrial volume index (LAVI) measured by 2D and also 3D echocardiography in 73 elderly patients with sinus rhythm. P wave dispersion was significantly correlated with 2D LAVI (*r* = 0.600), 3D systolic LAVI (*r* = 0.688) and diastolic LAVI (*r* = 0.566) [14]. A 3D systolic LAVI ≥ 25 mL/m2 separated patients with PWD ≥ 40 ms. An increased PWDIS associated with echocardiographic changes has also been described in other diseases. Tosu et al. [17], when studying the relationship between P wave dispersion in hypertensive patients, reported a significant correlation between P wave dispersion and left ventricular mass index (*r* = 0.412, *p* = 0.011) [16]. In addition, Chávez et al. [18] found a relationship between PWDIS and left ventricular hypertrophy [17].

The present study revealed a significant positive correlation between P wave dispersion and diastolic dysfunction grade (*r* = 0.354, *p* = 0.014) common in agreement with Akdemi et al. [19] study included 73 with diastolic dysfunction and 60 without diastolic dysfunction (control group) who found that P wave dispersion was significantly higher in in patients with diastolic dysfunction (53 ± 9 ms vs. 43 ± 9 ms; *p* < 0.01) [18]. Moreover, Donoiu et al. [20] study on 86 patients (47 patients with diastolic dysfunction and 39 without) reported that P wave dispersion was 62 ± 12 ms in patients with diastolic dysfunction and 49 ± 10 ms in those without (*p* < 0.01) [19].

The present study revealed a significant negative correlation in the present study between corrected P wave dispersion and aortic cusp sclerosis (*r* = − 0.330, *p* = 0.02) that come in agreement with Acar et al. [21] who found that aortic stiffness showed a negative correlation with PWDIS (*r* = − 0.52, *p* = 0.005) [6].

P wave dispersion was significantly higher among cases with Morris index > 0.04 common in line with the results detected by Lin et al. [22] that found that Morris index is statistically significant predictor of poor outcome among studied cases with PAF [21].

In the present study, P wave dispersion was statistically significant valid in differentiating recurrent PAF cases from non-recurrent cases with the best detected cutoff point from the curve that was 0.044 s yielding sensitivity of 93.5% and specificity 47.1% and also found to be a statistically significant predictor of PAF recurrence by multivariate analysis which is explained that prolonged intra- and interatrial conduction times and non-homogeneous propagation of sinus impulses are characteristic of PAF. An increase in PWD indicates heterogeneous intra- and interatrial conduction [23]. Previous studies have shown that PWD is significantly greater in patients with PAF than in controls, and that PWD ≥ 40 ms can differentiate patients with PAF with 83% sensitivity and 85% specificity [24–26].

## Conclusions

We concluded that a statistically significant association was detected between P wave dispersion and the following: older age, diabetic and hypertensive cases and also positive statistically significant correlation with LAD, left ventricle size and diastolic dysfunction grade. Mean corrected P wave dispersion and corrected QT interval were higher among cases using sotalol, ca channel blockers, among cases using nitrates and among cases with Morris index > 0.04. Higher mean value of corrected QT was associated with biphasic P v1 shape. Old age, female sex, P wave dispersion and QT wave dispersion are statistically significant predictors of PAF recurrence.

### Study limitations

- Small sample size.
- This study not including patients with ischemic heart disease presenting with PAF episodes.

## Data Availability

Data and material are available on a reasonable request from the author.

## Abbreviations

2D: Two-dimensional
AF: Atrial fibrillation
BPM: Beat per minute
CBC: Complete blood count
DC: Direct current
ECG: Electrocardiogram
IRB: Institution Research Board
IV: Intravenous
LAD: Left atrial diameter
LAVI: Left atrial volume index
PAF: Paroxysmal atrial fibrillation
PWD: P wave dispersion
ROC: Receiver operating characteristics curve
T2DM: Type 2 diabetes mellitus
